# Valorized deinking paper residue as fill material for geotechnical structures

**DOI:** 10.1038/s41598-021-01949-1

**Published:** 2021-11-16

**Authors:** Karmen Fifer Bizjak, Barbara Likar, Ana Mladenovič, Vesna Zalar Serjun

**Affiliations:** Slovenian National Building and Civil Engineering Institute, Dimičeva 12, 1000 Ljubljana, Slovenia

**Keywords:** Environmental sciences, Chemistry, Engineering, Materials science

## Abstract

This study introduces a novel geotechnical composite material comprising two types of fill material sourced from the paper industry—deinking paper sludge ash (DPSA) and deinking paper sludge (DPS). Five composites with different DPSA and DPS contents were investigated. Two composites were selected for further analyses. The technology and procedure for composite installation were implemented in field tests. The composites with 80% and 70% DPSA exhibited the elasticity required to withstand minor landslide slip deformations, in addition to achieving sufficiently high values of uniaxial compressive strength. The composites had a low maximum dry density value, which led to fewer settlements in the entire support structure. The enhanced shear characteristics can enable the construction of a thinner retaining wall. The delay between preparation and installation of the composites was further investigated. The field tests confirmed that the composites with 80% and 70% DPSA can be installed on the construction site 4 h and even 24 h after mixing. In 2018, a retaining wall structure with 70% DPSA and 30% DPS was successfully implemented near a railway line using conventional technology as followed-up research to the herein presented study. Results have been derived from work performed in the scope of the H2020 Paperchain project in which novel circular economy models centered on the valorization of the waste streams generated by the pulp and paper industry as secondary raw material for several resource-intensive sectors, including the construction sector, have been developed. Environmental benefits are savings in natural raw materials, reduction of landfill disposal as well as CO_2_ emission reduction.

## Introduction

The latest targeted policies and strategies implemented by the European Union to transition into a circular economy was issued in March 2020^1^, with the aim of accelerating the transformational changes required by the European Green Deal^2^. The circular economy paradigm warrants efficient and responsible use and management of resources as well as alternative materials instead of virgin ones. According to European Commission communication^1^, the global annual waste generation will increase to 70% by 2050. Consequently, the most viable alternative materials are different types of recycled waste. Large quantities of recycled waste can be accommodated in the construction sector, even if such materials are not environmentally inert, because it is possible to permanently immobilize hazardous components. In some cases, the characteristics of the new products are better than those of conventional, virgin materials. To this end, the construction sector can purchase raw materials from the pulp and paper manufacturing industry.

Globally, 420 million tons of paper and cardboard are produced every year^3^. Europe alone discards 11 million tons of paper waste per year ^4^. Approximately 70% of this waste originates from the production of de-inked recycled paper, the most voluminous being the different types of paper sludge and paper ash (generated during the incineration of paper sludge). The current management of these wastes, which involves primarily incineration or landfilling^4^, is far from preferable (prevent, reuse), according to the Waste Directive^5^.

The detailed state-of-the-art literature review of the recycling of pulp and paper industry wastes (wastepaper sludge and wastepaper sludge ash) is presented in Supplementary Tables 1 and 2 (SI1 and SI2). Table SI1 lists the various methods developed to valorize wastepaper sludge and wastepaper sludge ash. For example, the field of civil engineering has witnessed significant progress in recycling of wastepaper sludge and wastepaper sludge ash. Table SI2 reviews studies assessing the possible use of recycled paper sludge and paper sludge ash in geotechnical applications.

A few studies have used paper sludge and paper sludge ash as soil stabilizers for improving the performance of road construction materials and as controlled low-strength materials. The use of wastepaper sludge for the construction of low permeable barriers has also been evaluated. To the best of the authors’ knowledge, only Wu et al.^6^ have previously combined wastepaper sludge and wastepaper sludge ash to prepare a controlled low-strength geotechnical material. They studied a ternary to quinary component composite systems, each incorporating the conventional binder-Portland cement. They investigated composites at the laboratory level, e.g., paper fly ash as a partial substitution for cement (maximum 95% replacement) and paper bottom ash as a partial or total substitute for natural aggregate, with paper sludge as a fibrous admixture (maximum 10% addition).

The objective of our study was to develop a two-component geotechnical composite, comprising solely of deinking paper sludge ash (DPSA) and deinking paper sludge (DPS), with no addition of cement, and verify them on site (demonstration fields). Further, this study aims to identify the structure–property and process–structure relationships as they are the two key research fields in materials science and engineering. For this reason, this study includes a holistic set of analyses: microstructural (phase) characterization, assessing of chemical properties, and identification of geomechanical properties both on a laboratory, and on a field scale. The insufficient information about the detailed characteristics of such novel recycled composites points to the knowledge gap in this research area. The most important design parameters were the unconfined compressive strength, shear properties, elasticity, and frost resistance. Transportation of materials to the construction site could extend the time between mixing and installation considerably, which would significantly affect the geotechnical properties of the composite. The weakening of the properties with respect to the installation time has not been addressed in the literature. To evaluate the effect of this delay on the strength of the composite, we first prepared five compositions of DPSA and DPS and evaluated their geomechanical properties. Subsequently, the effect of the delay between mixing and installation of two composites with the best geomechanical properties was investigated on site. Finally, we assessed the potential environmental impact of the fill material.

We intended to use the final composite as a fill material for reinforcing a retaining wall structure near a railway line in Slovenia to stabilize landslides in that region. The followed up research was already successfully implemented in 2018, as reported by Fifer Bizjak et al.^7^ In this case, the geotechnical composite (hereinafter referred to as “fill material”) completely substituted conventional materials (aggregate and concrete) in the geotechnical structure. Research work was conducted in the scope of the H2020 Paperchain project, aimed to unlock the potential for a resource efficient model based on industrial symbiosis demonstrating the potential of the major waste streams generated by the pulp and paper industry as valuable secondary raw materials for construction, transport infrastructure applications and some other resource-intensive sectors.

## Materials and methods

The characteristics of the raw materials (deinking paper sludge ash and deinking paper sludge) together with the experimental program of the research work conducted are presented in the following subsections.

### Raw materials: deinking paper sludge ash and deinking paper sludge

Vipap Videm Krško d.d. is a paper mill company in Slovenia that produces newsprint and coated graphic paper using the deinking process. The annual production is 210,000 tons. The primary waste products from the deinking process are DPSA and DPS, with annual productions of DPSA 25,000 tons and DPS 67,000 tons, respectively. DPS was combusted in a boiler to generate electricity and heat energy; this yielded DPSA as the combustion residue from the boiler, which is composed of 90 wt% bottom ash and 10 wt.% fly ash. We used DPSA and DPS as raw materials (Fig. SI1). The European Waste Catalogue (EWC) classifies DPSA as 10 01 01 and DPS as 03 03 05^8^. Approximately 200 kg of each raw material was sampled (DPSA from silo, DPS from depot) from the VIPAP plant, according to SIST EN 14899^9^. After sampling, the DPSA sample was stored in airtight plastic barrels, while DPS was stored in plastic barrels in a climatic chamber (+ 4 °C).

#### Mechanical and physical properties of raw materials

Table 1 presents the mechanical and physical properties of DPSA and DPS.

**Table 1:** Mechanical and physical properties of DPSA and DPS.

Parameter	DPSA	DPS	Standard
Water content (w) (%)	0	47.5	SIST EN ISO 17892-1^10^
Particle density (ρ_s_) (Mg/m^3^)	2.64	2.15	SIST EN ISO 17892-3^11^
Optimum water content (*w*_*op*t_) (%)	51	57	SIST EN 13286-2/AC^12^
Maximum dry density (ρ_d,max_) (Mg/m^3^)	0.99	0.89	SIST EN 13286-2/AC^12^
Unconfined compressive strength after compaction (q_u_) (MPa)	0.45	0.22	SIST EN 13286-41^13^
Particle size distribution			
D_10_ (mm)	0.002	ND^a^	
D_50_ (mm)	0.04	ND^a^	
D_90_ (mm)	0.6	ND^a^	

^a^ND: particle size distribution analysis could not be performed, owing to the nature of DPS.

The particle size distribution of DPSA was measured using a CILAS 920 Particle Size Analyser (Cilas, Orléans, France). The results show that DPSA is a fine-grained material, with a median particle diameter of less than 63 µm.

#### Microstructural characterization of raw materials

The phase composition was characterized via X-ray powder diffraction (XRD) using an Empyrean diffractometer (PANalytical, Netherlands) with Cu–Kα radiation. Powder diffraction data were collected at a tube tension of 40 kV and a tube current of 45 mA, using a 2 Theta step size of 0.02° and a measurement time of 100 s per step. The results were analyzed using the Highscore (PANalytical, Netherlands) diffraction software with the Powder Diffraction File PDF-4+ (ICDD, USA) database as the reference data source. Prior to the XRD analysis, the samples were pulverized in an agate mortar to a size below 40 µm.

The major crystalline mineral phases of DPSA (Fig. SI2) are calcite, along with lime and portlandite. Trace quantities of quartz, talc, mellilite, anhydrite, illite/muscovite, and brownmillerite are also present. The elevated background in the XRD spectra in the 2θ range of 28°–38° suggests the presence of an amorphous phase.

Oprčkal et al.^14^ proved that DPSA is a hydraulically active material. In this study, to investigate the phase composition during the hydration of the ash over longer time intervals, samples were prepared into a paste by being mixed with an excess of demineralized water. They were then cured in a climatic chamber (at 90% RH and 22 °C) for 2, 7, 14, 56, 90 and 180 days. The samples were then analyzed via XRD at each curing time interval.

**Figure 1:**
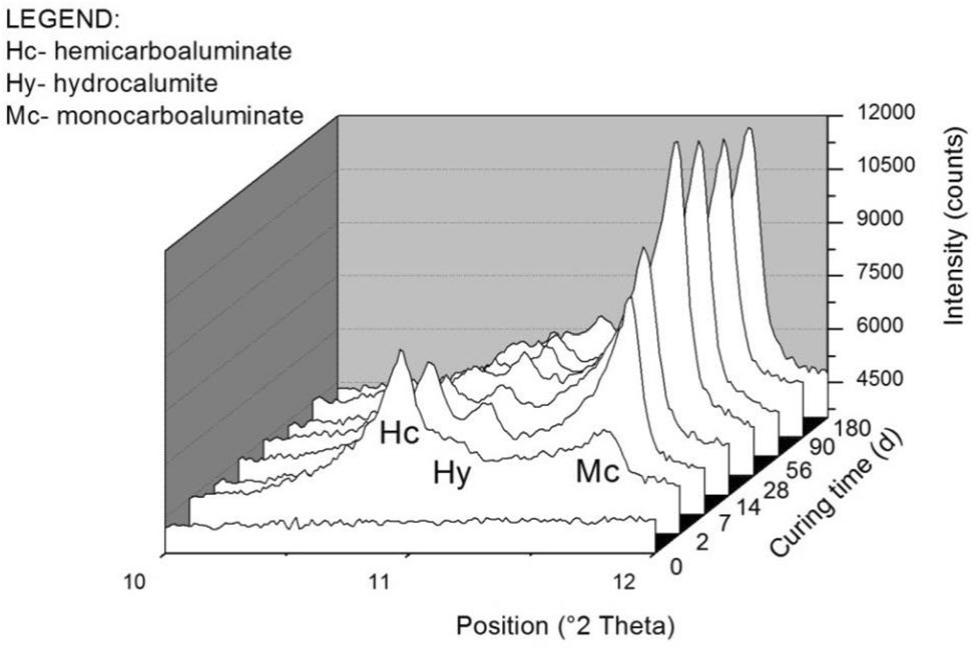
Sampled section of the XRD patterns of the DPSA paste at different curing time intervals. The graph indicates the most intense peaks of the newly formed mineral phases.

The results (Fig. 1) showed that DPSA reacts with water, resulting in the formation of hydration products, i.e., calcium aluminate hydrates (CAHs). After 2 days of hydration, hemicarboaluminate was found to be the most abundant product. After 28 days, the vast majority of hemicarboaluminate was converted to monocarboaluminate owing to the high quantity of calcium carbonate in the system. As the hydration time increased, hemicarboaluminate became depleted, the quantity of hydrocaluminate remained approximately constant, and monocarboaluminate became the prevalent hydration product from the DPSA paste. The hydraulic reactivity of DPSA was attributed to the reaction of amorphous aluminum and siliceous components with calcium hydroxide/oxide (portlandite/lime) in DPSA in the presence of water to form cementitious hydration products.

The XRD analysis of DPS revealed calcite and kaolinite to be the major crystalline phases and quartz and dolomite to be the minor phases (Fig. SI2). The presence of cellulose was confirmed based on two indistinct intensities in the range of two broad peaks: (110) at a 2θ of 19.9° and (020) at a 2θ of 22.1°, in combination with the appearance of the ($\overline{1 }$10) and (101) reflections at 2θ values of 12.8° and 14.9°, respectively.

Morphological, microstructural, and semiquantitative chemical analyses of DPSA and DPS were performed using a scanning electron microscope (SEM 5500 LV, JEOL, Japan) equipped with energy dispersive spectroscopy technology (EDS, Oxford instruments, UK). The conditions of the SEM/EDS analyses were low-vacuum mode, a chamber pressure of 20 Pa, and an accelerating voltage of 15 kV. Polished cross-sections of the samples were prepared in a form of resin mounted sample.

The result of the SEM/EDS analysis showed that a large proportion of the DPSA grains were irregular, composed of an amorphous groundmass with small, rounded pores (Fig. SI3). A systematic EDS point analysis of the amorphous phase revealed a primary composition of Si, Al, and Ca. Traces of K, Na, Mg, Cl, and Fe in different proportions were also found. The amorphous phase was finely intermixed with DPSA crystalline phases. A mineral phase consisting of Ba and S (baryte) was also observed. Relatively large grains of calcite were embedded with a fine-grained crystalline matrix. The analysis of DPS confirmed the presence of organic fibers, with a pronounced natural fiber structure in their longitudinal cross-section (Fig. SI3).

#### Chemical composition of raw materials

The bulk chemical compositions of DPSA and DPS were analyzed using a Thermo Scientific ARL PERFORM'X Spectrometer (Waltham, USA). A fused bead was prepared (lithium tetraborate with lithium metaborate in a ratio 1:1) with a mixture of the sample and flux in a mass ratio of 1:10 and then heated at 1025 °C. The results are presented in Table 2.

**Table 2:** Chemical compositions of DPSA and DPS.

Raw material	Parameter (wt%)										
	Si	Al	Fe	Ca	P	Mg	K	Na	Ti	S	LOI
DPSA	5.31	4.37	0.27	19.20	0.07	1.02	0.22	0.13	0.11	0.12	27.26
DPS	3.4	2.94	0.17	12.73	0.03	0.62	0.13	0.10	0.07	0.02	53.41

#### Leaching test of raw materials

Leaching tests were performed according to SIST EN 12457-2^15^. The contents of As, Ba, Cd, Cr, Cu, Hg, Mo, Ni, Pb, Sb, Se, and Zn were determined in accordance with SIST EN ISO 17294-2^16^ using coupled plasma mass spectrometry (ICP-MS, Agilent 7700x, Agilent Technologies, Tokyo, Japan). The uncertainty of these measurements was within ± 2%. The accuracy of the ICP-MS analysis result was verified by determining the elements from the standard reference material, SPS-SW1-Reference Material for Measurements of Elements in Surface Waters (Spectrapure Standards, Oslo, Norway) (Table SI3). The contents of Cl^−^ and SO_4_^2−^ in the leachate were defined using a UV/Vis HACH DR/2010 spectrophotometer (Loveland, CO, USA), with a measurement uncertainty of ± 5%. The accuracy of the determination of Cl^−^ and SO_4_^2−^ was verified by analyzing the standard reference material, Anions-Whole Volume (Merck KGaA, Darmstadt, Germany) (Table SI4). Table 3 presents the results of the leaching test.

**Table 3:** Concentrations of elements in the leachate from DPSA and DPS with the limiting values for inert materials set by the current European legislation^17^ and the basic parameters: pH and conductivity of the leachate.

Parameter (mg/kg d m)	DPSA	DPS	Limiting value for inert materials
As	< 0.01	0.035	0.5
Ba	34.9	0.23	20
Cd	< 0.01	< 0.002	0.04
Cr (total)	0.02	0.009	0.5
Cu	0.15	0.096	2
Hg	< 0.01	0.003	0.01
Mo	0.06	0.12	0.5
Ni	< 0.01	0.029	0.4
Pb	0.09	0.026	0.5
Sb	< 0.01	0.009	0.06
Se	< 0.01	< 0.003	0.1
Zn	0.63	0.11	4
Cl^−^	120	80	800
F^−^	4.5	9.1	10
SO_4_^2−^	20	470	1000
pH	12.7	7.1	
Conductivity (mS/cm)	9.7	1.2	

Table 3 shows that except Ba in the leachate of DPSA, no other potentially toxic element was present in a quantity exceeding the corresponding limiting value for inertness. Therefore, DPSA should be regarded as non-hazardous waste and DPS as inert waste.

### Experimental program

Experimental program comprised of: (1) laboratory tests on five different composites and (2) demonstration fields tests on two different composites.

#### Composite preparation

Five different composites of DPSA and DPS in different ratios were designed. The designations of composites were: D80/20 for 80 DPSA/20 DPS, D70/30 for 70 DPSA/30 DPS, D60/40 for 60 DPSA/40 DPS, D50/50 for 50 DPSA/50 DPS and D20/80 for 20 DPSA/80 DPS mixing ratio (wt% dry mass).

The raw materials were mixed in a 20-l Gostol planetary mixer (Gostol, Slovenia) until they formed a homogeneous mixture. The composites were compacted to the maximum dry density, as specified in SIST EN 13286-2/AC^12^, and cured at 90% RH and 22 °C in a climatic chamber.

#### Laboratory tests

##### Mineralogical analysis

The composites were analyzed using XRD after 50 days of curing, under the same diffractometer and measurement conditions as those described in “Microstructural characterization of raw materials”. CAHs, hemicarboaluminate, hydrocalumite, and monocarboaluminate, which are crucial for the immobilization of potential toxic elements, were used as indicative phases for the relative quantification of the newly formed crystalline mineral phases. The relative amounts of the hydration products were determined by the integrated intensities of peaks at d_100_ = 8.19 Å for hemicarboaluminate, d_100_ = 7.90 Å for hydrocalumite, and d_100_ = 7.57 Å for monocarboaluminate.

##### Geomechanical tests

The compressive strength of the composites was determined immediately after compaction and after 1, 4, 7, 28 and 50 days of curing, according to SIST EN 13286-41^13^. The vertical deformation at sample failure during the unconfined compressive test was also evaluated.

We performed a frost-resistance test according to the Slovenian National Technical Specification for Roads^18^, which requires 12 cycles of freezing and thawing at temperatures of − 20 °C and + 20 °C in a climatic chamber.

The shear characteristics (friction angle (f) and cohesion (c)) of the composites were tested after 7 days of curing and after the compaction test, according to SIST EN ISO 17892-10^19^.

To investigate the impact of the delay between preparation (mixing) and installation (compaction) of the composites on account of the transportation of materials from the production site to the construction site, additional tests were conducted on the two composites that achieved the best laboratory results (D80/20 and D70/30). The two samples for the time delay analysis were prepared and designated as defined in Table 4.

**Table 4:** Preparation of samples D80/20 and D70/30 and their designations to study the impact of time delay.

Composite	Sample preparation	
	Maximum water content (w_max_)/air condition (25 °C and 45% RH)	Optimal water content (w_opt_)/climatic chamber (22 °C and 90% RH)
D80/20	D80/20 w_max_	D80/20 w_opt_
D70/30	D70/30 w_max_	D70/30 w_opt_

All four samples for the time delay analysis were compacted immediately and after 2, 4, 8, and 24 h. The unconfined compressive strength of each sample was tested after 7 days according to SIST EN 13286-41^13^.

#### Preparation of demonstration test field

To simulate the actual conditions in which the composite would be installed and provide more realistic engineering parameters for the design of a fill material for a retaining wall structure, we chose D80/20 and D70/30 for valorization in the demonstration test field. Test fields with dimensions of 2 m × 2 m × 0.6 m were prepared from each composite at the Vipap Videm Krško’s facility (Table 5).

**Table 5:** Design parameters of D80/20 and D70/30 samples for installation in the demonstration test field.

Composite	Demonstration field	Time delay between mixing and compaction (h)	Moistening of mixture at installation
D80/20	TP 1.1	0	–
	TP 1.2	4	Sprayed with water to reach w_opt_ + 3%
	TP 1.3	24	remixed with water (0.03 m^3^ of water per 1.0 m^3^ of material)
D70/30	TP 2.1	0	–
	TP 2.2	4	Sprayed with water to reach w_opt_ + 3%
	TP 2.3	24	remixed with water (0.03 m^3^ of water per 1.0 m^3^ of material)

The composites were mixed in the field using a TERREX stirrer (Norwalk, CA, USA) for 15 min (Fig. SI4). We had planned to install the composites 4 and 24 h after mixing the raw materials. Until the installation, the samples were place on a stock and covered by a geosynthetic fabric. Using a BOMAG hand compactor (Boppard, Germany), the composites samples were installed and compacted in three layers (Fig. SI4), each with a thickness of 10–15 cm, to obtain at least 95% of the maximum dry density (ρ_**d,**max_).

The dry density (ρ_d_) and water content (w) of the composites installed in the demonstration test fields were measured on site using the TROXLER nuclear moisture density gauge (Ettenheim, Germany) according to Slovenian Technical Specifications (TSC)^20^, immediately after compaction and after 1 and 28 days. Dynamic deformation modules (E_vd_) on the upper layer were measured at the same time intervals, according to TSC^21^, using a lightweight deflectometer (ZORN, Stendal, Germany). To evaluate the potential environmental impact of the composite, the samples were collected from demonstration test fields TP 1.2 and TP 2.2 after 28 days of compaction with a drilling machine. These fields were chosen because fill materials for retaining wall structures must be prepared within 4 h before installation. Leaching was performed according to SIST EN 1744-3^22^. The leachate was analyzed via ICP-MS and UV–Vis spectrophotometry. The determined and certified values were close to each another (within ± 5% for spectrophotometry and ± 2% for ICP-MS). This confirmed the accuracy of the analytical procedure employed (Tables SI3 and SI4).

## Results and discussions

### Results of laboratory tests

#### Mineralogical analysis

**Figure 2:**
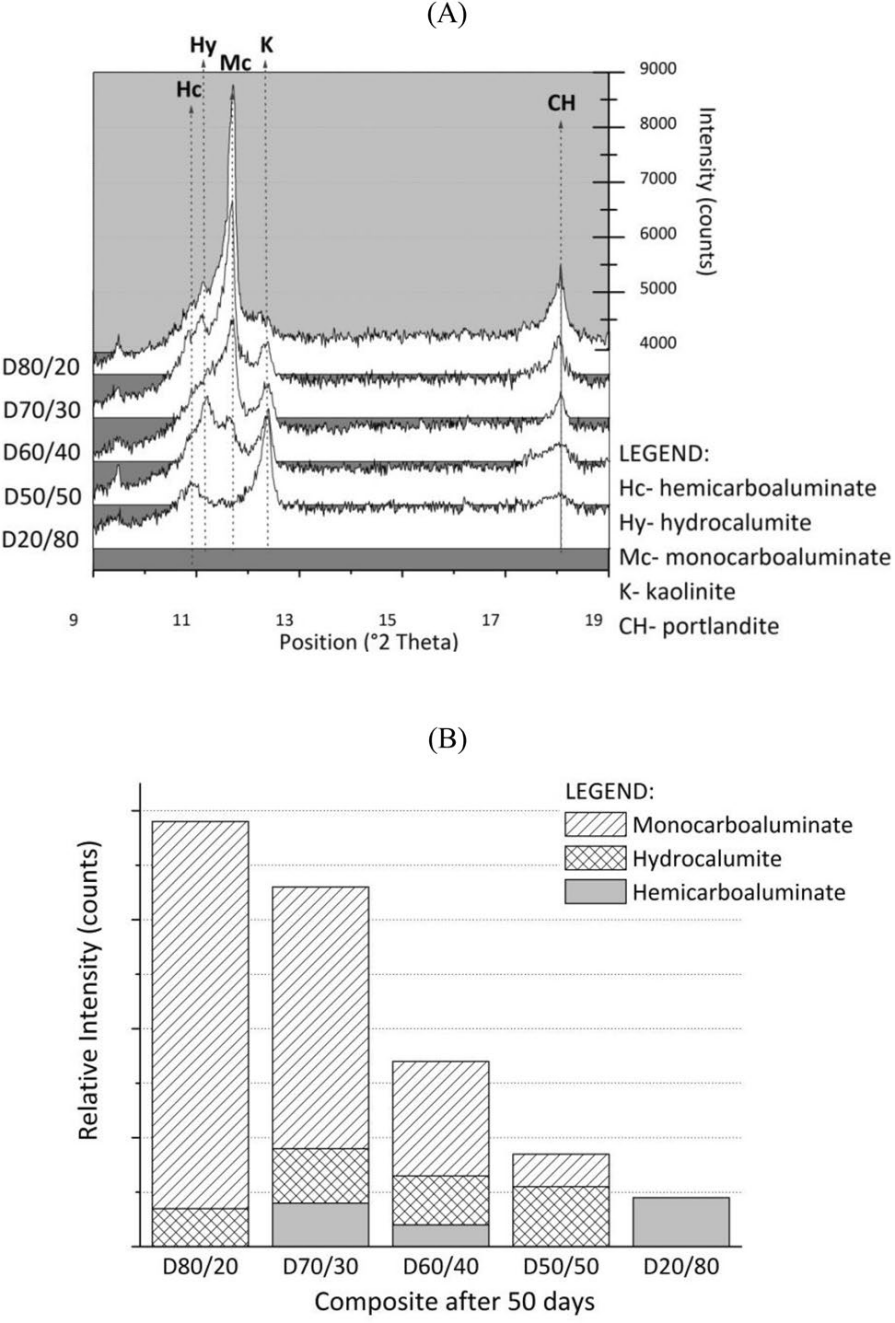
(**A**) Sections of the XRD patterns of the composites after 50 days of curing. Main peaks corresponding to hemicarboaluminate, hydrocalumite, and monocarboaluminate are indicated; (**B**) relative amounts of hydration products formed in the composites after 50 days of curing.

CAHs were the new mineral phases formed during the hydration of the investigated composites after 50 days of curing (Fig. 2A). Hydrocalumite and monocarboaluminate were identified in composites D80/20, D70/30 D60/40, and D50/50. Hemicarboaluminate was also detected in D70/30 and D60/40, while hemicarboaluminate was the only hydration product in D20/80. The high quantity of calcium carbonate in the system resulted in the conversion of hemi- to monocarbonate CAH, which is also more stable than CAH phases with (OH^−^). Mineral phases such as calcite, kaolinite, quartz, dolomite, talc, illite/muscovite, and portlandite were present in all the investigated composites. These phases represent the constituents of the raw materials of the composites.

The relative quantification of the crystalline mineral phases (Fig. 2B) revealed that D80/20 had the highest quantity of hydration products and D20/80 the lowest. The quantity of hydration products in the composites decreased as the amount of DPSA reduced, which was attributed to the hydraulic characteristics of DPSA.

#### Standard Proctor tests (SPP)

**Figure 3:**
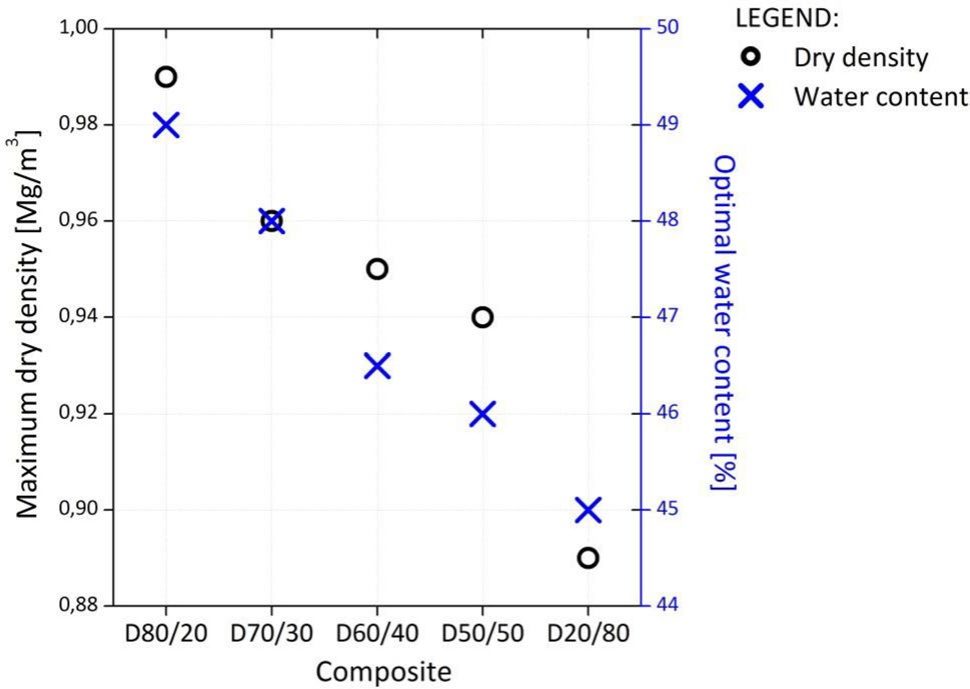
Maximum dry density and optimal water content of the investigated composites.

The results of the SPP showed that the quantity of DPSA influenced the optimal water content (w_opt_) and the maximal dry density (ρ_max_) of the composites. The optimal water content increased from 45% for the D20/80 to 49.5% for D80/20 as the percentage of DPSA increased (Fig. 3). This was attributed to the higher moisture content of DPS and to the DPSA hydraulic characteristic. The maximal dry density followed a similar trend, increasing from 0.89 Mg/m^3^ for D20/80 to 0.99 Mg/m^3^ for D80/20.

A comparison of the proposed composites with the virgin aggregate material shows that the former had a significantly lower dry density. The average maximum dry density of the virgin aggregate (e.g., limestone) material was 2.2 mg/m^3^, whereas that of the composites was 0.89–0.99 mg/m^3^. This will enable the application of the composites on terrains with precarious geotechnical conditions because the fill material will causes fewer settlements than the conventional material will. This is an important attribute of the investigated composites as a backfill material for geotechnical structures in regions with low-bearing-capacity foundations.

#### Unconfined compressive strength (q_u_)

**Figure 4:**
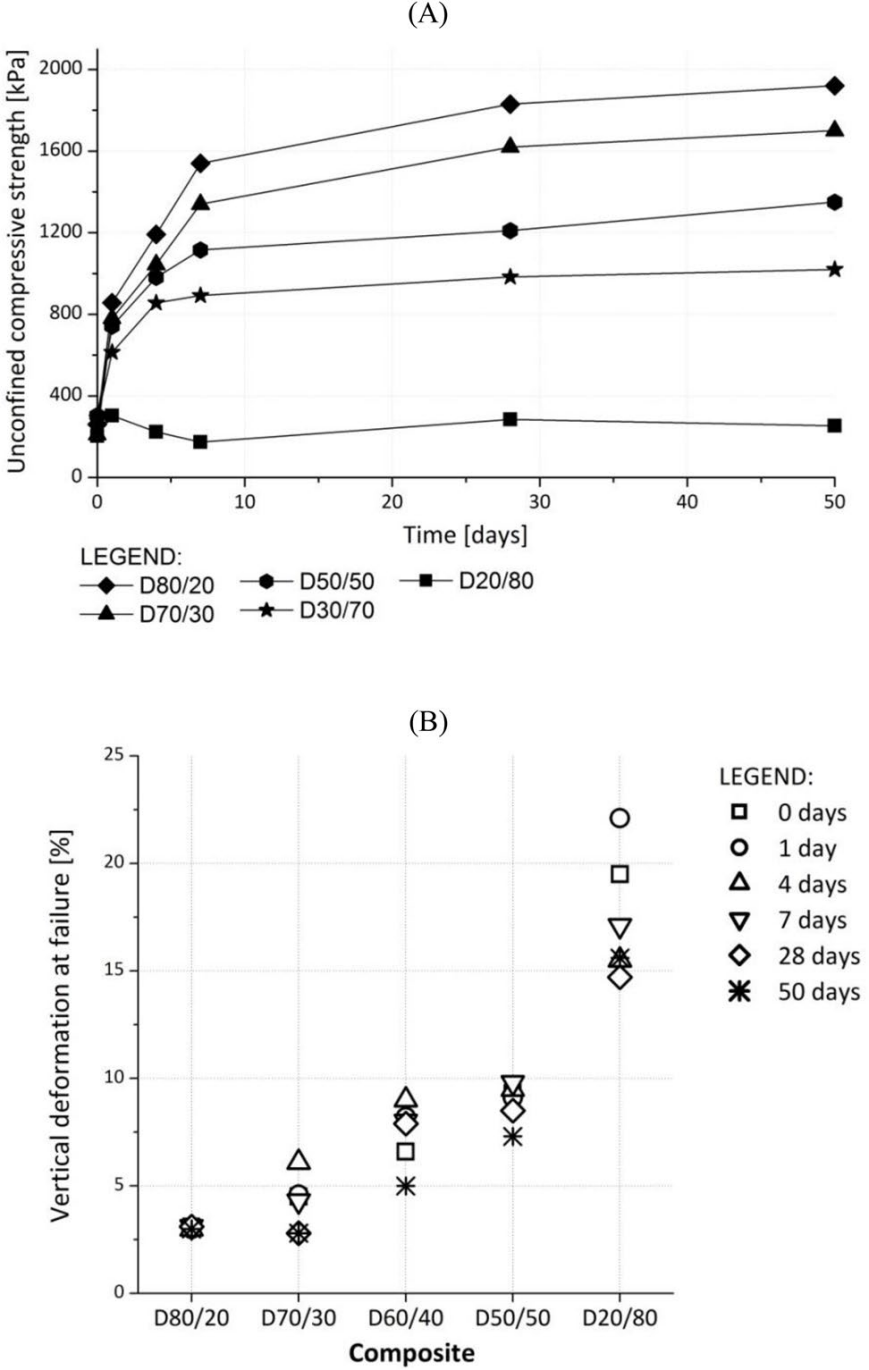
(**A**) Unconfined compressive test results and (**B**) vertical deformation at sample failure during unconfined compressive test of investigated composites at different time intervals.

Higher quantities of DPSA in the composites signify an increase in the unconfined compressive strength value (Fig. 4A). *q*_*u*_ increases with the curing time interval, except in D20/80, because it contains the lowest quantity of DPSA and the highest quantity of DPS. The tests performed immediately after compaction yielded similar values of q_u_ (0.2–0.3 MPa), irrespective of the composition. Composites with higher percentages of DPSA and a lower percentage of DPS exhibited higher *q*_*u*_ values after 1 day of curing, accounting for the higher quantities of hydration products formed in these composites.

The maximum vertical deformations at failure (e_A_) after the unconfined compressive test are illustrated in Fig. 4B. The vertical deformation decreased as the quantity of DPSA in the composite decreased from 3 to 23%. In D80/20, this was manifested in a rapid breakage with small elastic deformations, whereas in D20/80, it appears as a slower breakage with a large deformation. The higher quantity of DPS allowed for larger elastic deformations of the composites before failure.

The vertical deformation in most of the composites depended on the curing time (Fig. 4B). While curing time had a negligible influence on e_A_ in the case of D80/20, for D20/80, e_A_ was between 15% (50 d cured sample) and 22% (1 days cured sample). For the other composites, e_A_ increased for up to 4 or 7 days and subsequently decreased. This implies that the failure mode of the composites changed from plastic to brittle between the first and fourth/seventh days of curing. The fill materials must possess sufficient elasticity as well as strength to prevent landslide movements.

#### Frost resistance

**Figure 5:**
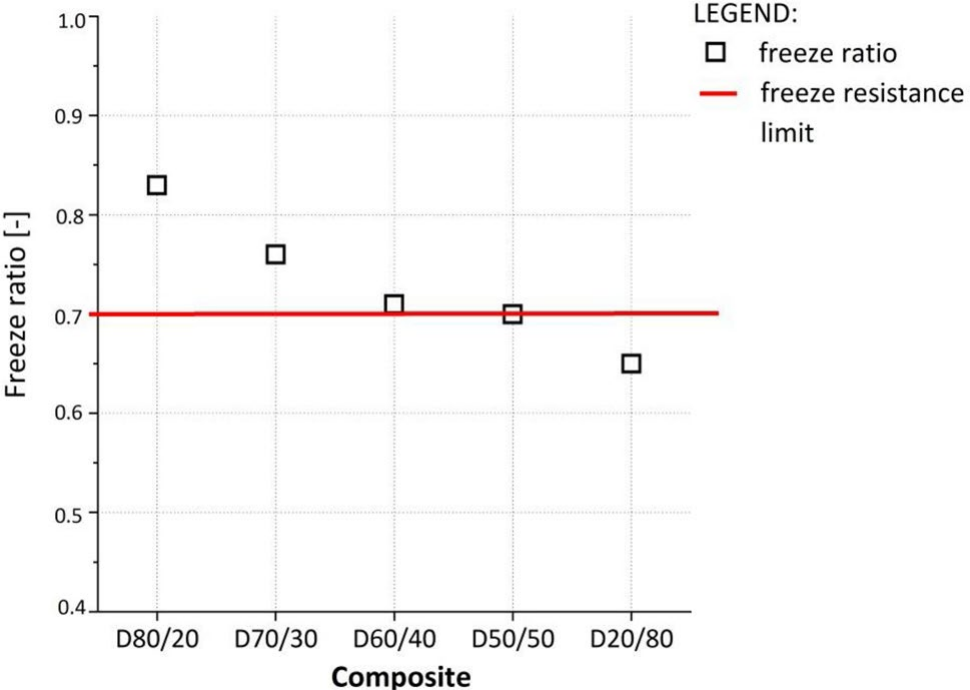
Frost resistance test results for investigated composites.

According to TSC 06.320^18^, a freeze ratio (i.e., the ratio of the unconfined compressive strength of samples exposed to the freeze/thaw cycles and untreated samples cured in the climatic chamber) of more than 0.7 was set. The composites with 40% DPS (D60/40) or less (D70/30, D80/20) comply with this requirement (Fig. 5) and can be defined as freeze/thaw resistant. The results showed that these composites could be used as a fill material even in layers less than 1 m below the surface. This depth is also the overall frost depth (frost line) in Slovenia.

#### Shear properties

**Figure 6:**
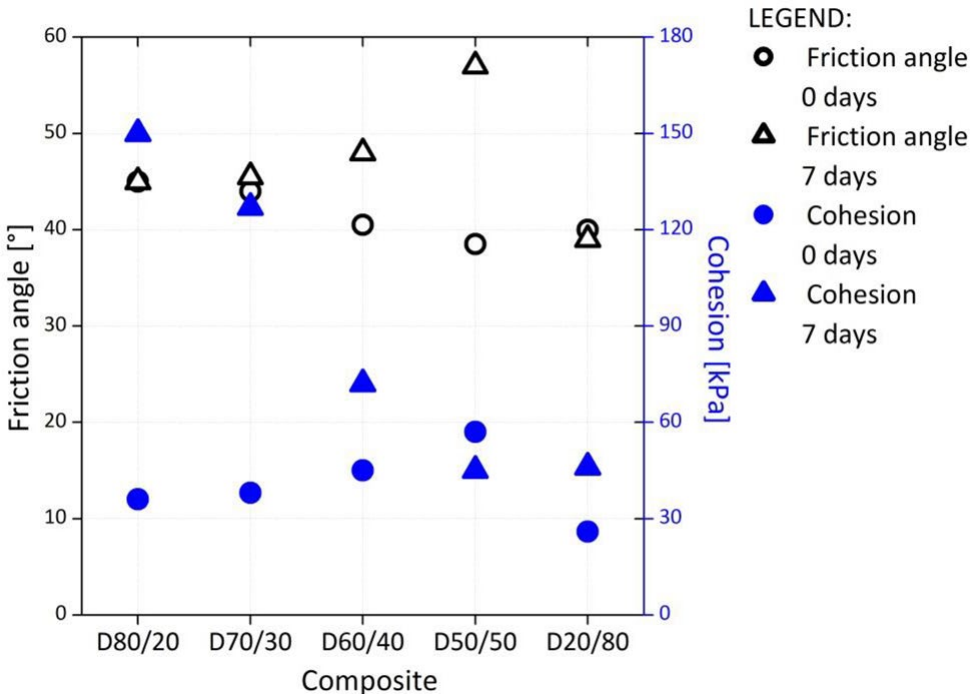
Shear test results of the investigated composites immediately after compaction and after 7 days of curing.

The values of the shear parameters—friction angle (f) and cohesion (c)—increased with the amount of DPSA (Fig. 6). The friction angle of the virgin limestone aggregate materials conventionally used to construct retaining walls was approximately 30°; this material exhibited no cohesion. The shear properties of the composites exhibited higher values (Fig. 6). The investigated composites can enable construction of a thinner yet resilient retaining wall, which is especially important for railway projects in mountainous regions, where railway lines are narrow, and adequate space for larger geotechnical structures is unavailable.

### Impact of time delay between mixing and compaction

**Figure 7:**
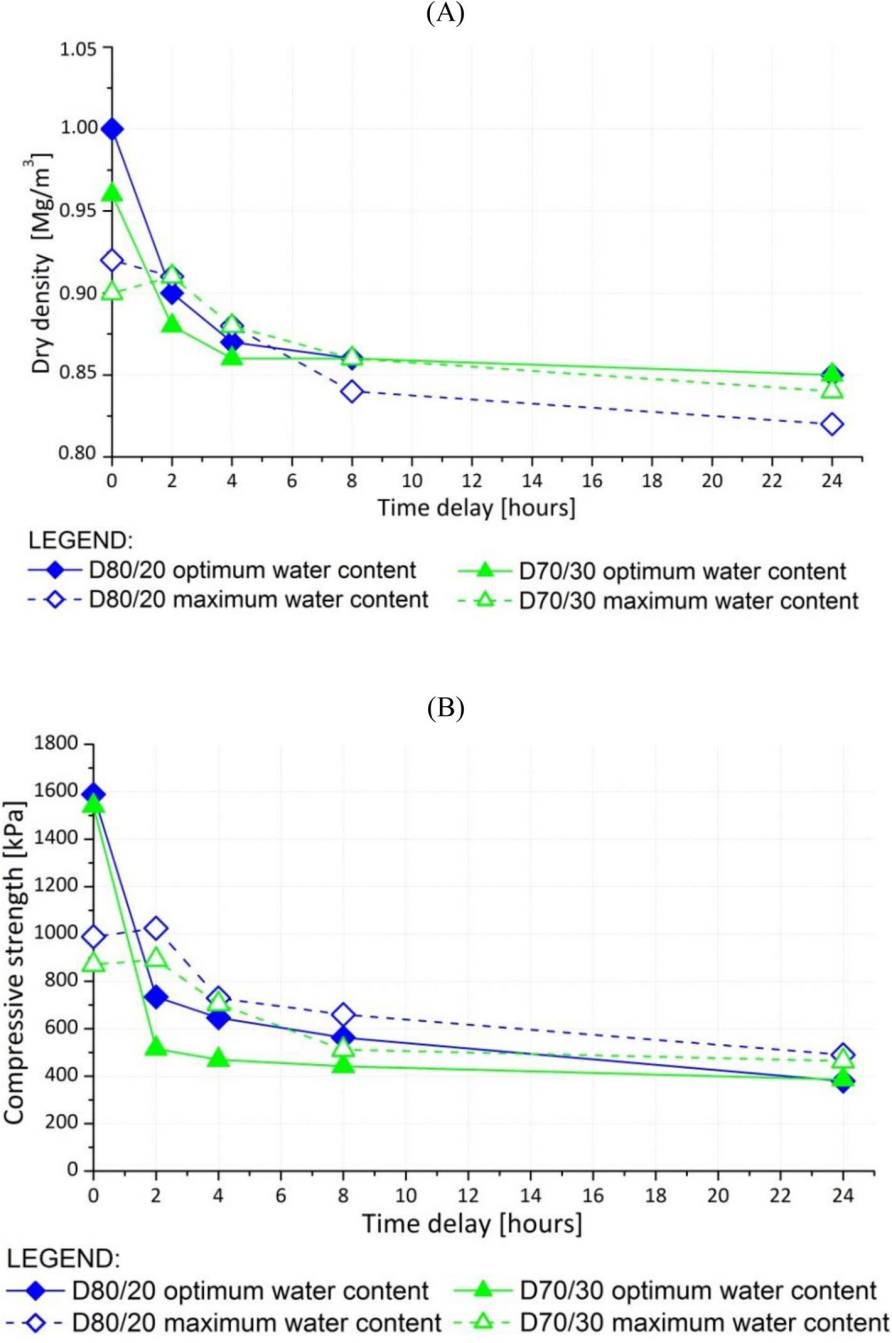
(**A**) Dry density and (**B**) unconfined compressive strength of D80/20 and D70/30 as a function of time delay between mixing and compaction.

The investigation of the impact of time delay between mixing and compaction of D80/20 and D70/30 on the geotechnical composite (Fig. 7A) revealed that the maximum dry density (ρ_d,max_) decreased with an increasing delay. The decrease in ρ_d,max_ was more pronounced in samples compacted at the optimal water content (w_opt_). The results of the uniaxial compressive tests (*q*_*u*_) (Fig. 7B) showed higher values for the samples compacted immediately after mixing at w_opt_, and 8–9% lower values for those compacted immediately after mixing at the maximum water content (w_max_). After 4 h, samples compacted at w_max_ exhibited higher uniaxial compressive strengths than those compacted at w_opt_. The values of the uniaxial compressive strength were nearly identical for all the samples after 24 h.

The highest values of unconfined compressive strength (*q*_*u*_) and ρ_d,max_ were defined for the samples prepared at w_opt_ and compacted immediately after mixing (Fig. 7B). With regard to the samples compacted 4 h after mixing, a higher *q*_*u*_ was measured for the composites prepared at the maximum water content (w_max_). The hydration process dried the composites, and after 4 h, it resulted in a lower *q*_*u*_ for the mixture with a lower water content. After 24 h, the values of ρ_d,max_ and *q*_*u*_ became constant. Based on these results, we concluded that the composite compacted immediately after preparation must be moistened to w_opt_. The composite compacted 4 h after preparation owing to the transportation delay must be moistened to w_max_.

### Demonstration field test

According to the results of the laboratory tests, D80/20 and D70/30 exhibited the most suitable geomechanical properties; its unconfined compressive strength (*q*_*u*_) was comparable to that of the conventional material, and it exhibited satisfactory elastic behavior compared with both concrete and compacted virgin aggregate. Laboratory tests showed that the composites can be compacted properly even 24 h after their preparation. Figure SI5 presents the demonstration fields.

Table 6 lists the results of the SPP for D80/20 and D70/30 installed in the demonstration field test.

**Table 6:** Results of the SPP for samples installed in the demonstration fields.

Demonstration field	SPP	
	ρ_d.maximum_ (mg/m^3^)	w_opt_ (%)
TP 1.1	0.99	49
TP 1.2	0.87	49
TP 1.3	0.85	49
TP 2.1	0.96	48
TP 2.2	0.86	48
TP 2.3	0.85	48

### Characteristics of the installed composites

**Figure 8:**
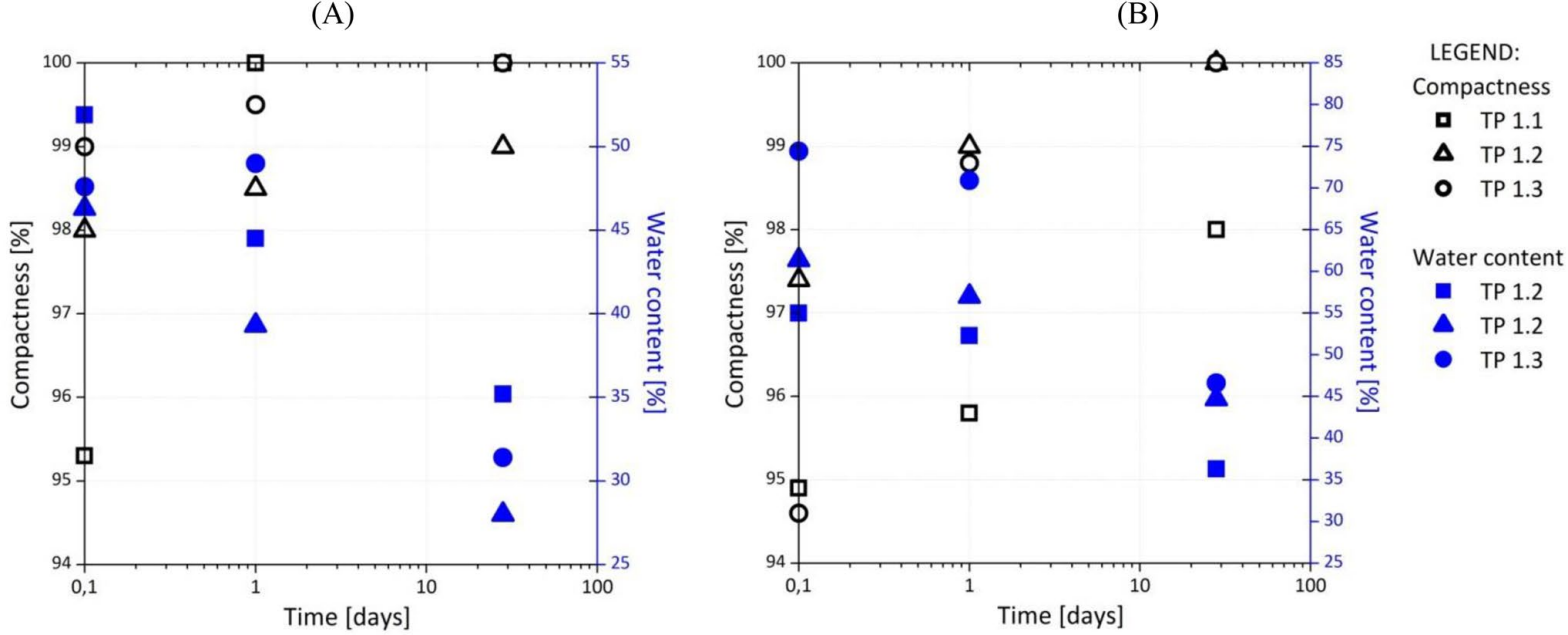
(**A**) Compactness and water content of D80/20 and (**B**) of D70/30 demonstration test fields.

Figure 8 presents the results of the compactness test for D80/20 and D70/30, respectively. The percentage of compactness was calculated by dividing the dry density (ρ_d_) with the maximum dry density (ρ_d,max_). According to TSC 05.413^23^, the compactness of composites to be used as fill materials for the construction of retaining wall structures must be at least 95% of the SPP. The compactness increased with time for all the composites and was greater than 95%, except for D70/30, which was installed 24 h after mixing. The compactness of D70/30 increased with time and reached its limiting value after 1 day.

Meanwhile, we used more water during installation than the optimal water content determined via the SPP for all the demonstration test fields samples (+ 3%), because we estimated the need for an additional quantity of water for the chemical reaction of DPSA (the hydraulic reaction of DPSA consumes 3% water).

**Figure 9:**
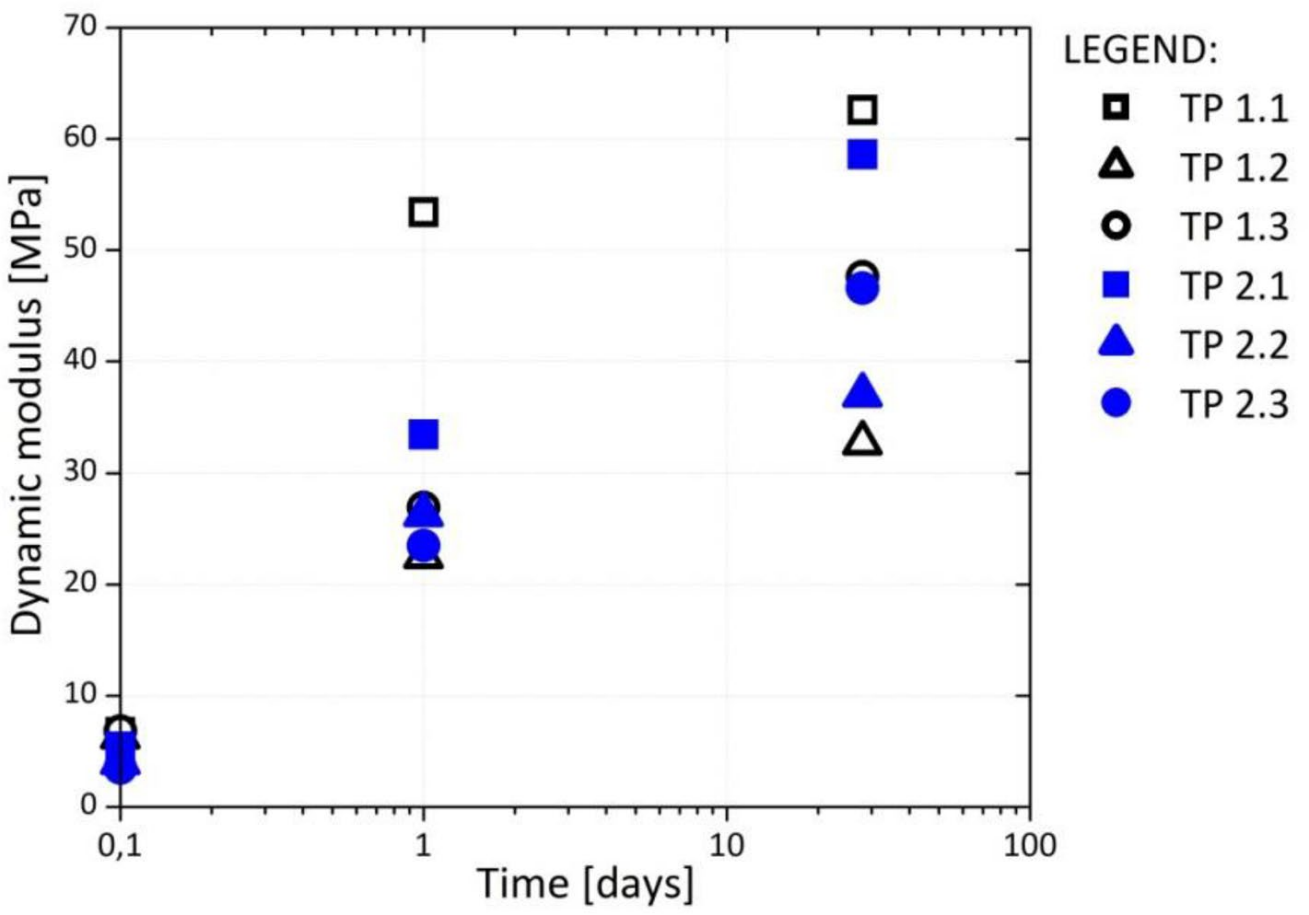
Dynamic modulus vs. time for the demonstration tests fields.

The dynamic deformation module (E_vd_) was measured to be 3.0–8.0 MPa (Fig. 9). The bearing capacity increased with time owing to the curing, and after 28 days, it surpassed 30 MPa. According to TSC 05.413^23^, E_vd_ for a fill material must be at least 15 MPa. All composites met this criterion within 1 day.

The DPSA content impacts E_vd_. Composites with a higher DPSA content exhibited a higher E_vd_. The time of compaction is also an important parameter determining E_vd_. The composites compacted 4 h after mixing showed a lower E_vd_ than those installed immediately after preparation. The opposite trend was observed for the composites installed 24 h after mixing. These composites showed a higher E_vd_ than those installed after 4 h. This finding was ascribed to the additional water content (without additional water and mixing, the composite cannot be installed amid the already insufficiently low water content).

The field test results indicate that the proposed technology is appropriate for installing the composite as a fill material for retaining walls. The composites must be infused with a higher water content as determined through the SPP. Based on the empirical observation, the hydraulic reaction of DPSA consumes 3% water.

#### Leaching tests results

Table 7 lists the results of the tank leaching test. The potential toxic elements (PTE) concentrations in all the leachates from the composites in both demonstration test fields were within the limits for inert materials, as per current European legislation^17^. The concentrations of Ba exceeded the limit in raw DPSA but were within the limit for D80/20 (12 mg/kg d m) and D70/30 (16 mg/kg d m). Thus, D80/20 and D70/30, which were used for constructing TP 1.2 and TP 2.2, do not pose any environmental risk.

**Table 7:** Results of leaching tests from composites from the demonstration fields.

Parameter (mg/kg d m)	Composite		Limiting value for inert materials
	D80/20	D70/20	
	Demonstration field		
	TP 1.2	TP 2.2	
As	< 0.02	< 0.02	0.5
Ba	12	16	20
Cd	< 0.005	< 0.005	0.04
Cr (total)	0.041	0.025	0.5
Cu	1.6	1.9	2.0
Hg	< 0.001	< 0.001	0.01
Mo	0.085	< 0.070	0.5
Ni	0.014	0.023	0.4
Pb	0.066	0.056	0.5
Sb	< 0.006	< 0.006	0.06
Se	< 0.01	0.011	0.1
Zn	< 0.10	< 0.1	4.0
Cl^−^	41.8	46.5	800
F^−^	2.2	1.8	10
SO_4_^2−^	35.4	28.0	1000

## Conclusions

A series of laboratory experiments were performed to explore the feasibility of composites with different compositions of DPSA and DPS to be used as a filling material for retaining walls in landslide-prone regions. Based on the laboratory results, two composites, one with 80% DPSA and the other with 70% DPSA, were selected for further laboratory and field valorization to simulate the installation and provide more representative engineering parameters for the design purposes. The results of these tests are key to determining the method of mixing, transportation, and installation of the fill material on the construction site and to assess the maximum time delay between the material production and the installation.

Results are summarized as follow.

1. The low maximum dry density values of the composites compared with those the conventional virgin fill material enables their application in terrains with low-bearing-capacity foundations.
2. The high elasticity, frost resistance, and high strength of the proposed composites are important for the construction of landslide-resistant geotechnical structures.
3. The high shear characteristics of the composites compared with those of the conventional virgin fill material reflect the ability of the former to allow for the construction of thinner wall structures to support the unstable slope behind it.
4. Composites can be installed and compacted even 24 h after their preparation, without any significant degradation of the mechanical properties.

The field test results demonstrated the composites with 80% and 70% DPSA comply with the recommendations regarding fill materials for the construction of geotechnical structures and are environmentally compatible. As of 2018, using conventional technology, we had, on the basis of the results presented herein, already constructed a retaining wall structure using fill materials with the 70% DPSA and 30% DPS composites, at a site near a railway line in southeast Slovenia. This represents a practical example of the circular economy between the paper and construction industries which has increased environmental sustainability. Onsite field monitoring of the pilot retaining wall structure is still ongoing.

## Supplementary Information

Supplementary Information.
