# Supplementary material for: Valorized deinking paper residue as fill material for geotechnical structures
Source: Sci Rep. 2021 Nov 16;11:22363. doi: 10.1038/s41598-021-01949-1 (PMC8595652; doi:10.1038/s41598-021-01949-1)
Supplement: Supplementary file 1 — Supplementary Information. [file 41598_2021_1949_MOESM1_ESM.pdf]

Valorized deinking paper residue as fill material for geotechnical structures

Karmen Fifer Bizjak[a], Barbara Likar[a], Ana Mladenovič[a], Vesna Zalar Serjun[a,*]

[a] Slovenian National Building and Civil Engineering Institute, Dimičeva 12, 1000 Ljubljana, Slovenia

*Corresponding author: V. Zalar Serjun

E-mail address: vesna.zalar@zag.si; Telephone: +386 1 2804341; Fax: +386 1 2804484;

ORCID: 0000-0003-1658-2466

SUPPLEMENTARY INFORMATION

Supplementary Figures

Deinking paper sludge ash (DPSA) Deinking paper sludge (DPS)

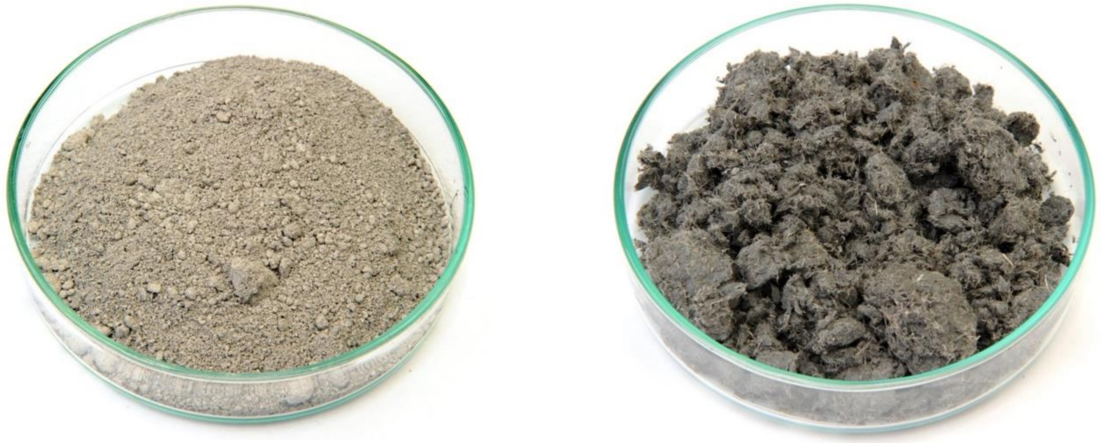

Fig. SI1 Appearance of raw materials used for the preparation of the geotechnical composites.

Deinking paper sludge ash (DPSA) Deinking paper sludge (DPS)

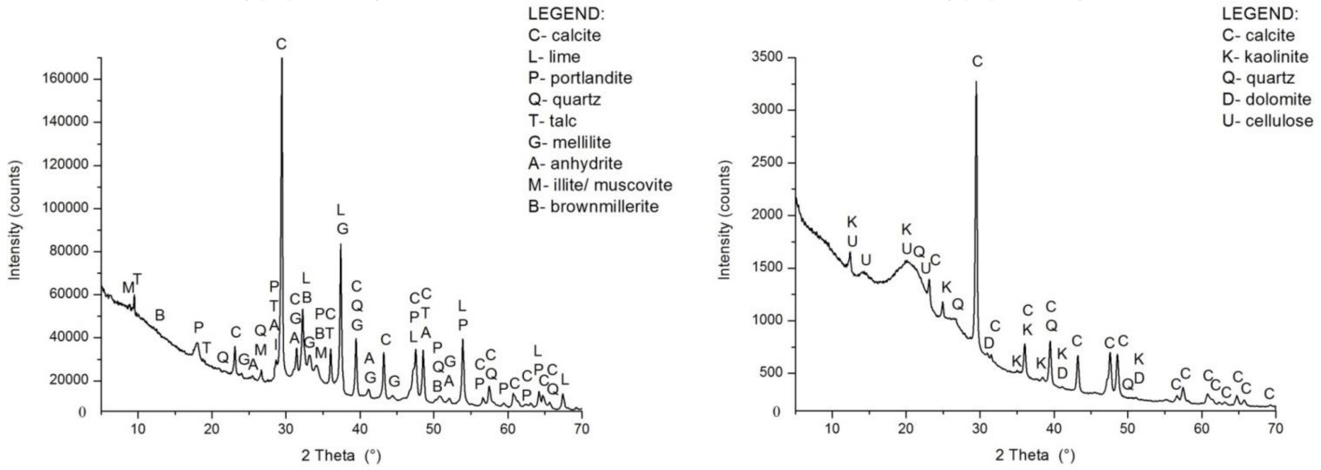

Fig. SI2 XRD patterns of raw materials used for the preparation of the geotechnical composites.

Deinking paper sludge ash (DPSA) Deinking paper sludge (DPS)

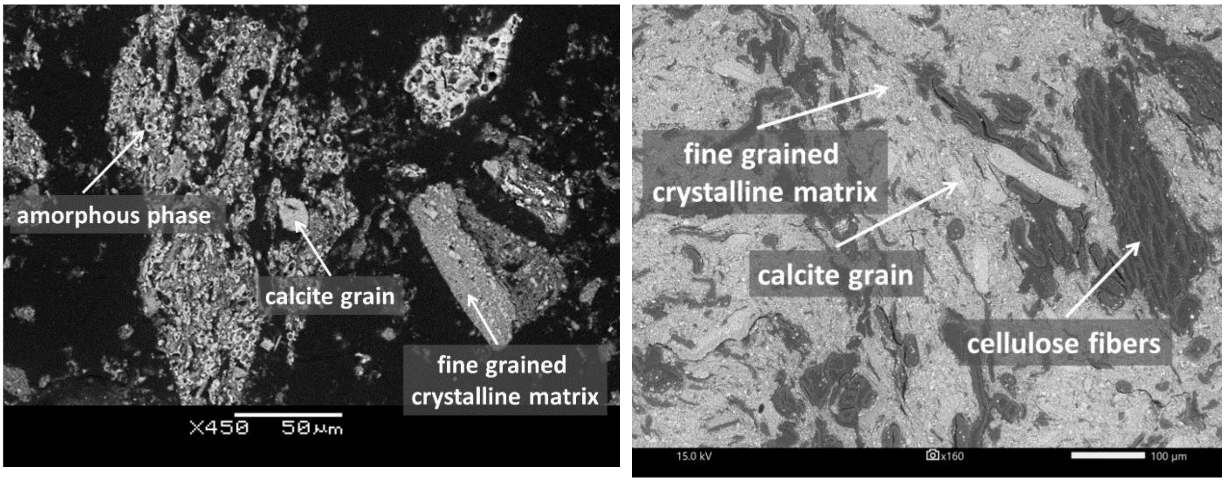

Fig. SI3 SEM micrographs of the raw materials used for the preparation of the geotechnical composites

(A) (B)

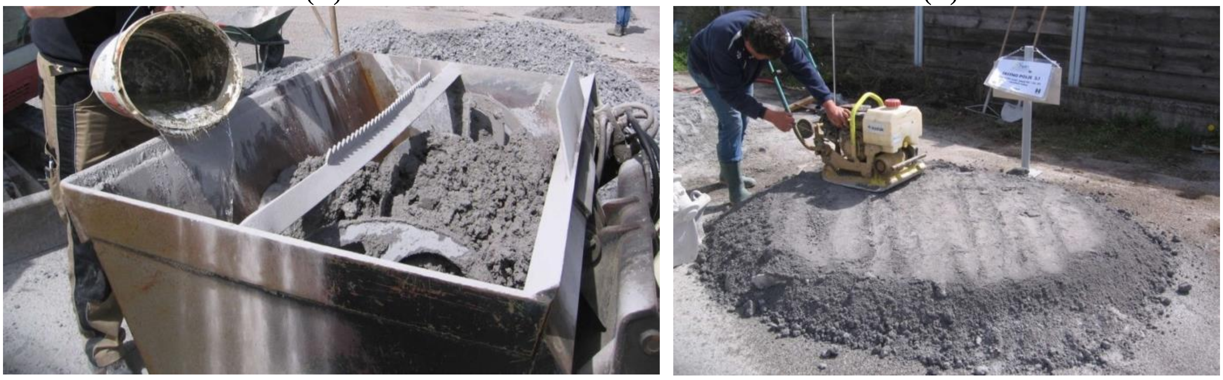

Fig. SI4 (A) Mixing of DPSA and DPS for preparation of D80/20 demonstration test field TP 1.2; **(B)** compaction of D70/30 (layer 2) on the TP 2.1 demonstration test field.

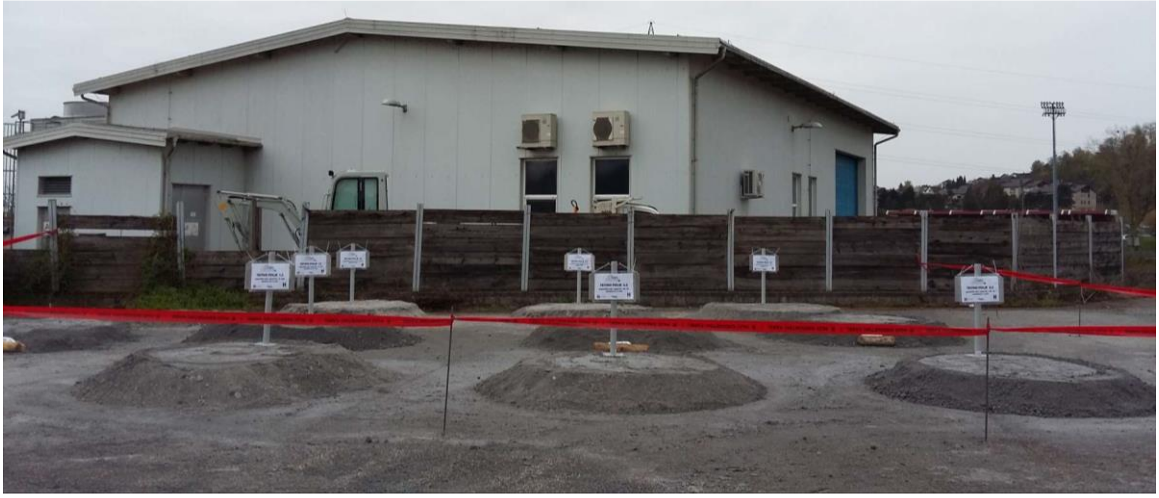

Fig. SI5 Demonstration fields

Supplementary Tables

Table SI1 Summary of the different researched fields of the application of wastepaper sludge and wastepaper sludge ash.

Research field	Intended use	Reference
		**Wastepaper sludge**
Papermaking material	Reuse in a paper mill	Tofani, G., et al., 2020. Alternative filler recovery from paper waste stream. Waste Biomass Valor. https://doi.org/10.1007/s12649-020-01011-7
Energy recovery techniques	Waste biomass feedstock for thermochemical methods (pyrolysis, direct liquefaction, steam reforming, anaerobic digestion, and gasification); biogas production (anaerobic digestion)	Zhang, Z., et al., 2015. Simultaneous recovery of organic and inorganic content of paper deinking residue through low temperature microwave-assisted pyrolysis. Environ. sci. Technol. 49, 2398–2404. https://doi.org/10.1021/es505249w<br><br>Steffen, F., et al., 2017. Valorization of waste streams from deinked pulp mills through anaerobic digestion of deinking sludge. Bioresources, 12, 4547–4566. https://doi.org/10.15376/BIORES.12.3.4547-4566<br><br>Mendez, A., et al., 2014. Biochar from pyrolysis of deinking paper sludge and its use in the treatment of a nickel polluted soil. J. Anal. Appl. Pyrol. 107, 46–52. https://doi.org/10.1016/j.jaap.2014.02.001<br><br>Ivan, D., Asta, K., Mika, H., 2015. Deinking sludge utilization possibilities: Technical, economic,

		and environmental assessments. LUT Scientific and Expertise Publications/Research Reports. http://urn.fi/URN:NBN:fi-fe201505269106
Soil amendment and a plant nutrient carrier	Addition to top-soil in agriculture	Phillips, V.R., et al., 1997. The use of paper-mill sludges on agricultural land. Biores. Technol. 60, 73–80. https://doi.org/10.1016/S0960-8524(97)00006-0
Absorbent material for metals	Metal-ion removal from water	Calace, N., et al., 2003. Metal ion removal from water by sorption on paper mill sludge. Chemosphere, 51, 797–803. https://doi.org/10.1016/S0045-6535(02)00864-0
Manufacture of pozzolanic material	Supplementary cementitious material	Naik, T.R., Friberg, T.S., Chun, Y.-M., 2004. Use of pulp and paper mill residual solids in production of cellucrete. Cem. Concr. Res. 34, 1229–1234. http://dx.doi.org/10.1016/j.cemconres.2003.12.013.<br><br>Garcia, R., et al., 2008. The pozzolanic properties of paper sludge waste. Constr. build. mater. 22, 1484–1490. https://doi.org/10.1016/j.conbuildmat.2007.03.033<br><br>Frías, M., Rodríguez, O., Rojas M.I.S., 2015. Paper sludge, an environmentally sound alternative source of MK-based cementitious materials. A review. Constr. Build. Mater. 74, 37–48. https://doi.org/10.1016/j.conbuildmat.2014.10.007
Obtaining hydraulic material	Alite and belite;<br>clinker production	Pelisser, C., et al. 2020. Alite and belite obtained from the sludge of a paper recycling process. J. Mater. Cycles Waste Manag. 22, 1237–1248. https://doi.org/10.1007/s10163-020-01016-3

		Simão, L., et al., 2017. Waste-containing clinkers: Valorization of alternative mineral sources from pulp and paper mill. Process Safety Environ. 109, 106–116. https://doi.org/10.1016/j.psep.2017.03.038
Ceramic industry	Building bricks preparation	de Azevedo, A.R.G., et al., 2019. Characterizing the paper industry sludge for environmentally-safe disposal. Waste Manag. 95, 43–52. https://doi.org/10.1016/j.wasman.2019.06.001<br><br>Singh, S.K., et al., 2018. Sustainable utilization of deinking paper mill sludge for the manufacture of building bricks. J. Cleaner Prod. 204, 321–333. https://doi.org/10.1016/j.jclepro.2018.09.028
Manufacture of fiberboard	Board materials	Geng, X., Zhang, S.Y., Deng. J., 2007. Characteristics of paper mill sludge and its utilization for the manufacture of medium density fiberboard. Wood Fiber Sci. 39, 345–351.
Production of asphalt mixture	Additive for bituminous mixture; substitution of mineral filler in asphalt	Mari, E.L., et al., 2009. Paper mill sludge as fiber additive for asphalt road pavement. Philipp. J. Sci. 138, 29–36.<br><br>Wei, C.J., et al., 2020. Microscopic analysis and mechanical properties of recycled paper mill sludge modified asphalt mixture using granite and limestone aggregates. Constr. Build. Mater. 243, 118172. https://doi.org/10.1016/j.conbuildmat.2020.118172
Lightweight (sintered) aggregates for pre-stressed concrete production	Co-sintering of paper sludge with H_3BO_3	Hu, S.C., et al., 2012. Lowered temperature resource recycling of paper sludge using a co-melting technology. Bioresources 7, 2766–2783.

	**Wastepaper ash**	
Slag former	Replacement of primary lime in the argon oxygen decarburization (AOD) stainless steelmaking process	Hu, X., et al. 2020. Utilization of fly ash and waste lime from pulp and paper mills in the argon oxygen decarburization process. J. Clean. Prod. 261, 121182. https://doi.org/10.1016/j.jclepro.2020.121182
Improvement of water quality	Production of high ion exchange capacity material (microporous material)	Ishimoto, H., Origuchi, T., Yasuda, M., 2000. Use of Papermaking Sludge as New Material. J. Mater. Civ. Eng. 12. https://doi.org/10.1061/(ASCE)0899-1561(2000)12:4(310)
Soil remediation	The remediation of contaminated soil via an immobilization procedure	Oprčkal, P., et al., 2020. Remediation of contaminated soil by red mud and paper ash. J. Clean. Prod. 256, 120440. https://doi.org/10.1016/j.jclepro.2020.120440
Agriculture	Calcium amendment in agriculture	Muse, J.K., Mitchell, C.C., 1995. Paper mill boiler ash and lime by-products as soil liming materials. Agron. J. 87, 432–438.
Cement-based materials / Usage as supplementary cementitious material	Mineral addition; cementitious material; Mortars and concrete production by the usage of SCM	Ishimoto, H., Origuchi, T., Yasuda, M., 2000. Use of Papermaking Sludge as New Material. J. Mater. Civ. Eng. 12. https://doi.org/10.1061/(ASCE)0899-1561(2000)12:4(310)<br><br>Martínez-Lage, M., et al., 2016. Concretes and mortars with wastepaper industry: Biomass ash and dregs. J. Environ. Manag. 181, 863–873. https://doi.org/10.1016/j.jenvman.2016.06.052<br><br>Segui, P., Aubert, J.E., Husson, B., Measson, M., 2012. Characterization of wastepaper sludge

		ash for its valorization as a component of hydraulic binders. Appl. Clay Sci. 57, 79–85. https://doi.org/10.1016/j.clay.2012.01.007<br><br>Segui P., Aubert, J.E., Husson, B., Measson, M., 2013. Valorization of wastepaper sludge ash as main component of hydraulic road binder. Waste Biomass Valor. 4, 297–307. https://doi.org/10.1007/s12649-012-9155-1<br><br>Mavroulidou, M., Awoliyi, S., 2018. A study on the potential use of paper sludge ash in concrete with glass aggregate. Waste Manage. Res. 36. https://doi.org/10.1177/0734242X18801196<br><br>Azrizal, M., et al., 2019. The properties of wastepaper sludge ash and its generic applications. J. Phys. Conf. Ser. 1349 012087. https://doi.org/10.1088/1742-6596/1349/1/012087
Clinker production	Hydraulic binder production	Simão, L., et al., 2017, Waste-containing clinkers: Valorization of alternative mineral sources from pulp and paper mill. Process Safety Environ. 109, 106–116. https://doi.org/10.1016/j.psep.2017.03.038
Glass-ceramics	Component for the preparation of glass-ceramics material	Toya, T., et al., 2006. Preparation and properties of glass-ceramics from kaolin clay refining waste (Kira) and paper sludge ash. Ceram Int. 32,789–96. http://dx.doi.org/10.1016/j.ceramint.2005.06.008
Manufacture of boards	Preparation of calcium silicate board	Chen, M. et al., 2019. Recycling of paper sludge powder for achieving sustainable and energy-

	materials	saving building materials. Constr. Build. Mater. 229, 116874. https://doi.org/10.1016/j.conbuildmat.2019.116874
Geopolymer materials	Precursor substitution in geopolymer composites	Mamat, N., Kusbiantoro, A., Rahman, N., 2018. Hydrochloric acid-based pre-treatment on paper mill sludge ash as an alternative source material for geopolymer. Mater. Today: Proc. 5, 21825–21831. https://doi.org/10.1016/j.matpr.2018.07.038<br><br>Cherian, C., Siddiqua, S., 2019. Pulp and paper mill fly ash: A review. Sustainability. 11, 4394. https://doi.org/10.3390/su11164394
Aggregate production	Light-weight aggregate for various applications	Perumal, P., Ganesh, G.M., Santhi, A.S., 2012. A review on artificial aggregates. Int. J. Earth Sci. Eng. 5, 540–546.

Table SI2 Summary of studies assessing the application of wastepaper sludge and wastepaper sludge ash in geotechnical application

Investigated material	Scope	Composite preparation	Composite characterization	Reference
**wastepaper sludge**				
– paper mill sludge	to study the chemical and geotechnical properties	sampling from the outlet over a period of 3 days	chemical and geotechnical properties, leachability of trace elements	Kuokkanen, T., et al., 2008. Chemical and leaching properties of paper mill sludge. Chem. Speciat. Bioavailab., 20, 111-122. https://doi.org/10.3184/095422908X324480
– paper sludge	to study the suitability of its use for *in situ* applications	sampled paper sludge, taken at the end of the dewatering process in different periods of the activity of the plant	laboratory analyses; geotechnical, physical, chemical analysis and leaching tests	Boni, M.R., D'Aprile, L., De Casa G., 2004. Environmental quality of primary paper sludge. J. Hazard. Mater. 108, 125-128. https://doi.org/10.1016/j.jhazmat.2003.11.017
**As a low permeable barrier**:				
– paper mill sludge	to investigate its beneficial reuse as landfill cover and bottom liner material in municipal waste landfills	numerical modeling	two-dimensional finite element analyses	Balkaya, M. 2019. Assessment of the geotechnical aspect of the use of paper mill sludge as landfill cover and bottom liner material. Desalination Water Treat. 172, 70–77. https://doi.org/10.5004/dwt.2019.25134

– paper pulp sludge –mixture with Class C fly Ash and three different polymers	to optimize a design that would be suitable as a hydraulic barrier in the form of a landfill liner	paper pulp sludge admixed with fly ash and polymers; compaction test	organic content, atterberg limits, consolidation, california, bearing ratio, shear strength, gas permeability, and liquid permeability	Slim, G.I., et al., 2016. Optimization of polymer-amended fly ash and paper pulp millings mixture for alternative landfill liner. procedia engineering. 145, 312–318. https://doi.org/10.1016/j.proeng.2016.04.079.
– different paper mill sludges	to find its beneficial use as the impermeable barrier in landfill cover	compaction test	consolidation, strength, permeability, long term infiltration tests	Moo-Young, H.K., Jr., Zimmie, T.F., 1997. Waste minimization and re-use of paper sludges in landfill covers: A case study. Waste Manag. Res. 15, 593–605.
– paper sludge	to test for its chemical, stability and sealing properties	sampling from five paper millsduring a period of 1 year	chemical and geomechanical analyses, biodegradability and sealing tests	Zule, J., Likon, M., Černec, F., 2007. Chemical properties and biodegradability of waste paper mill sludges to be used for landfill covering. Waste manage. Res. 25, 538–546. https://doi-org.nukweb.nuk.uni-lj.si/10.1177/0734242X07079188
– deinking by product	to identify the biodegradation parameters that could influence the long-term behavior of DBP covers	construction of biodegradation cells, compaction in three layers	biodegradation, leachate analysis, evolution of gas, hydraulic and geomechanical properties	Panarotto, C.T., Cabral, A.R., Lefebvre, G., 2005. Environmental, geotechnical, and hydraulic behaviour of a cellulose-rich by-product used as alternative cover material. J. Environ. Eng. Sci. 4, 123–138. https://doi.org/10.1139/s04-062

– deinking by product	to evaluate the field behavior and performance of a cover with capillary barrier effect to control water percolation	experimental plot constructed on the landfill site; cover system consists of a seepage control layer where a layer of DBP was compacted on top of a capillary barrier	the distribution of precipitation, calculations of degrees of saturation, suction	Abdolahzadeh, A.M., Lacroix Vachon, B., Cabral, A.R., 2011. Evaluation of the effectiveness of a cover with capillary barrier effect to control percolation into a waste disposal facility. Can. Geotech. J. 48, 996–1009. https://doi.org/10.1139/t11-017
– paper sludge composite mixtures with various additives (wood ash, fly ash, cement)	to evaluate the suitability of Slovenian wastepaper sludge as hydraulic barrier in landfill covers	samples of various paper sludges and composite mixtures; Cylindrical and prismatic samples; two field test cells	water content; uniaxial compressive and shear strength; hydraulic conductivity; electric conductivity.	Kortnik, J., Černec, F., Hrast, K., 2008. Paper sludge layer as low permeability barrier on waste landfills. Soil Sediment Contam. 17, 381–392. https://doi-org.nukweb.nuk.uni-lj.si/10.1080/15320380802146586
– pulp and paper sludge	to identify whether compacted pulp sludge would perform equally well as a barrier layer as compacted clay and a geomembrane	compacted pulp sludge; barrier plot installation; a 1.4-ha on-site ash landfill closed by using a compacted sludge barrier system	exfiltrate quantity and quality, tracer applications, soil water content	Ham, V.M., et al., 2009. Pulp and paper sludge as a barrier layer in landfill closure: A new opportunity. Pulp Pap. Can. 110, 25–30.
**For soli stabilization:**				

– paper mill sludge<br>–5, 10 and 15% addition to different soil samples	to study the possibility of its use for stabilization of village road sub-base	soil admixed with sludge; standard Proctor test	Atterberg limits, compaction properties, California bearing ratio value	Talukdar, D.K., 2015. A study of paper mill lime sludge for stabilization of village road sub-base. Int. J. Adv. Res. Technol. 5, 739–746.
– paper mill sludge<br>– replacement of 2, 4, 6, 8 and 10% of laterite soil	to investigate the effect on the strength, stability, and durability of laterite soil	soil admixed with ash; compaction test	specific gravity, Atterberg's limit, compressive strength, California bearing ratio test, durability test	Akshatha, B.A., Jain, A., 2020. Evaluation of laterite soil stabilized using waste paper sludge. Int. J. Res. Sci. Innovation 7, 132–136.
– paper mill sludge<br>– addition of 14 and 17% to the different clays	to assess its effectiveness as an alternative to lime or cement for clay stabilization	clays intermixed with sludge and compacted; two different curing methods of specimens	uniaxial compression, water retention and volumetric behavior.	Mavroulidou, M., 2018. Use of waste paper sludge ash as a calcium-based stabiliser for clay soils. Waste Manage. Res. 36, 1066–1072. https://doi.org/10.1177/0734242X18804043.
– paper mill sludge<br>– addition of 2, 5 and 8% to the marine soil	to investigate the properties of marine soil intermixed with DPS (5% of DPS was the optimal percentage to stabilize the marine soil)	marine soil intermixed with DPS, compaction test.	measurements of Atterberg Limits Test; TriaxialTest.	Mansor and Ishamuddin; http://122.129.122.220/bitstream/123456789/2115/1/ DIGES%20AKADEMIK%20PSA.pdf (accessed 20 October 2020)

**Wastepaper ash**				
**For soli stabilization:**				
– wastepaper sludge ash – 2, 4, 6, 8, 10, 12 and 14% intermixed with clay soil	to investigate and to show the potential use of wastepaper sludge ash as an additive to stabilize a clay soil	cylindrical specimens, Proctor test	development of compressive strength at different curing periods, California bearing ratio (soaked and unsoaked condition)	Khalid, N., et al., 2012. Clay soil stabilized using waste paper sludge ash (WPSA) mixtures. Electron. J. Geotech. Eng. 12, 1215–1225.
- paper sludge ash - 4, 6, 8, 10, 12% addition to the black cotton soil	to assess its effect of on engineering behaviors of black cotton soils (soil stabilization)	soil admixed with ash; compaction test	liquid and plastic limit tests, compressive strength, California bearing ratio	Dharan, R.B., 2016. Effect of waste paper sludge ash on engineering behaviors of black cotton soils. Int. J. Earth Sci. Eng., 9, 188–191.
- paper sludge ash - 50% addition to the contaminated soil; 25% admixing with red mud admixed with contaminated soil	to critically valorize ash and a mixture of ash and red mud for the remediation of contaminated soil via an immobilization procedure in which a	soil admixed with ash; compaction test	mineralogical composition, immobilization efficiency, compressive strength, water permeability	Oprčkal, P., et al., 2020. Remediation of contaminated soil by red mud and paper ash. J. Clean. Prod., 256, 120440. https://doi.org/10.1016/j.jclepro.2020.120440

	chemically inert geotechnical composite is obtained			
– paper sludge ash<br>– soil material from unpaved road intermixed with different fillers	to investigate the effect of two types of bioash as stabilization binders, studied using the Partial least squares structural equation modeling.	samples prepared in the laboratory (cube molds), road stabilization pilot case (4% cement, 9% bioash)	modeling; field tests: bearing capacity	Sarkkinen, M., et al., 2018. Effect of biomass fly ashes as road stabilisation binder. Road Mater. Pavement Des. 19, 239–251, https://doi.org/10.1080/14680629.2016.1235508
- paper mill ash<br>- 20 and 30% of ash in combination with road material; field- 30% of ash addition	to analyze the different sections of gravel road stabilized with paper ash regarding stiffness and environmental impact	ash gravel mixtures - laboratory and field tests	compression strength- also after six weeks of hardening and after 12 freeze-thawing cycles, infiltration capacity, lysimeters, bearing capacity	Vestin, J., et al., 2012. Fly ash as a road construction material, In: Arm, M., Vandecasteele, C., Heynen, J., Suer, P., Lind, B. (Eds.), WASCON, 1–8.
- paper sludge ash<br>- ash, lime, soil mixtures (three different soils)	- to assess the performance of mixes in terms of load bearing	laboratory test of different mixture; compaction test; two test road sections	environmental impact, engineering performance	Zhou, H., Smith, D.W., Sego, D.C. 2000. Characterization and use of pulp mill fly ash and lime by products as road construction amendments. Can. J. Civ. Eng. 27, 581–593.

	capacity, serviceability, and compaction			
– paper mill fly ash –20 and 30% by weight of fly ash in combination with road base material	to present and compare the field performance of road sections stabilized // Considerable improvement of the road performance can be achieved	based on the laboratory results, a mix with 30% of fly ash and 70% of road base material used for the field test- two road test sections	field performance of the stabilized road was inspected and investigated through evaluation of bearing capacity and strength, infiltration capacity	Arm, M., et al., 2014. Pulp mill fly ash for stabilization of low-volume unpaved forest roads - field performance. Can. J. Civ. Eng. 41, 955–963. https://doi.org/10.1139/cjce-2014-0030
**For Controlled Low Strength Material:**				
- paper sludge ash; 5, 10, 20 and 30% as a substitute for Portland cement	to investigate the possibility of using the recycled aggregate and SCM, without using a PC, to produce CLSM for various applications	mixing; cube shaped specimens	compressive strength, strength development	Ridzuan, A.R.M., et al., 2011. Strength assessment of controlled low strength materials (CLSM) utilizing recycled concrete aggregate and wastepaper sludge ash, IEEE Colloquium on Humanities, Science and Engineering, Penang, 2011, pp. 208–211, https://doi.org/10.1109/CHUSER.2011.6163718.
- paper sludge ash	to determine the strength of CLSM using PSA in the CLSM mix as	recycle concrete aggregate: binder = 1:1 and 1:2; different quantity of	slump test, compressive strength	Azmi, A.,N., Fauzi, M.,A., Nor, M.,D., Ridzuan, A., R., M., Arshad, M., F., 2015. Production of controlled low strength material utilizing waste paper sludge ash and recycled

	replacement of Portland cement	replacement of cement; mixing; cube shaped specimens		aggregate concrete, in: 3rd International Conference on Civil and Environmental Engineering for Sustainability, IConCEES 2015 – Melaka, Malaysia, MATEC Web of Conferences, 47, 01011, p. 1–8. https://doi.org/10.1051/matecconf/20164701011. EDP Sciences.
**Combination of wastepaper sludge and wastepaper ash**				
**For Controlled Low Strength Material:**				
– paper mill sludge (as fibrous admixture, paper mill fly ash (as SCM), paper mill bottom ash (as substitute for fine aggregate)	to explore the feasibility of utilizing solid wastes/byproducts from paper mills in CLSM	pre-blending, mixing, cylindrical specimens	flowability tests, mechanical performance	Wu, H., et al., 2016. Utilization of solid wastes/byproducts from paper mills in controlled low strength material (CLSM). Constr. Build. Mater. 118, 155–163. https://doi.org/10.1016/j.conbuildmat.2016.05.005

Table SI3 Concentrations of elements in standard reference material SPS-SW1 (reference material for measurements of elements in surface waters), Spectrapure Standards, Oslo, Norway). Concentrations of elements were determined by ICP-MS. The results represent the mean concentration obtained from three parallel samples ± standard deviation.

Element	SPS-SW1 Certified (μg L^{-1})	SPS-SW1 Determined (μg L^{-1})
As	10.0 ± 0.1	9.9 ± 0.2
Ba	50 ± 1	49 ± 1
Cd	0.50 ± 0.01	0.50 ± 0.01
Cr	2.00 ± 0.02	2.01 ± 0.04
Cu	20 ± 1	20.1 ± 0.4
Mo	10.0 ± 0.1	10.09 ± 0.2
Ni	10.0 ± 0.1	9.9 ± 0.2
Pb	5.0 ± 0.1	4.91 ± 0.01
Se	2.00 ± 0.02	2.03 ± 0.04
Zn	20*	18.7 ± 0.4

* Informative value

Table SI4 Concentrations of chlorides fluorides and sulfates in standard reference material Anions – Whole Volume (Merck KGaA, Darmstadt, Germany), determined by spectrophotometry. The results represent the mean concentration obtained from three parallel samples ± standard deviation.

Anions	Anions – Whole Volume Certified (mg L^{-1})	Anions – Whole Volume Determined (mg L^{-1})
Cl^-	95.0 ± 9.50	92.0 ± 5.0
F^-	1.17 ± 0.117	1.05 ± 0.06
SO_4^{2-}	44.3 ± 4.43	41.0 ± 2.0
